# Macular neovascularization in eyes with pachydrusen

**DOI:** 10.1038/s41598-021-87083-4

**Published:** 2021-04-05

**Authors:** Kelvin Yi Chong Teo, Kai Xiong Cheong, Ricardo Ong, Haslina Hamzah, Yasuo Yanagi, Tien Yin Wong, Usha Chakravarthy, Chui Ming Gemmy Cheung

**Affiliations:** 1 Singapore National Eye Centre, 11 Third Hospital Ave, Singapore, 168751 Singapore; 2 Singapore Eye Research Institute, Singapore 20 College Road Discovery Tower, Level 6 The Academia, Singapore, 169856 Singapore; 3 University of Sydney, Camperdown, NSW 2006 Australia; 4 Department of Ophthalmology, Asahikawa Medical University, Hokkaido, Japan; 5 Department of Ophthalmology, Yong Loo Lin School of Medicine, National University of Singapore, Singapore, 1E Kent Ridge, NUHS Tower Block, Level 7, Singapore, 119228 Singapore; 6 Ophthalmology Academic Clinical Program, Duke-NUS Graduate Medical School, 8 College Road, Singapore, 169857 Singapore; 7 School of Medicine, Dentistry and Biomedical Sciences, Queens University Belfast, 97 Lisburn Rd, Belfast, BT9 7BL UK

**Keywords:** Retinal diseases, Macular degeneration

## Abstract

The natural history and clinical significance of pachydrusen is unclear. This study aims to compare the longitudinal changes of eyes with pachydrusen and soft drusen and progression to exudative macular neovascularisation (MNV). Patients with a diagnosis of MNV in one eye only and the fellow eye was selected as the study eye. Study eyes were required to have pachydrusen or soft drusen on fundus photographs and follow up of at least 2 years or until exudative MNV occurred. Systematic grading was performed at baseline and change in drusen area and onset of exudative MNV recorded over the period of follow up. A total of 75 eyes from 75 patients (29 with pachydrusen and 46 with soft drusen) were included. There was no difference in the rate of progression to exudative MNV in the soft and pachydrusen groups (13.3% versus 24.1%, p = 0.38). Pachydrusen, as compared to soft drusen, was associated with polypoidal choroidal vasculopathy subtype (85.7% versus 16.7%, p < 0.01) and the location of exudation was co-localised with soft drusen but not with pachydrusen. There was a higher rate of increase in soft drusen area compared to pachydrusen area (27.7 ± 31.9%/year versus 8.7 ± 12.4%/year respectively, p < 0.01). We found no difference in the proportion of eyes that developed exudative MNV in this study however characterisation of drusen evolution patterns revealed a strong association with exudative MNV subtype.

## Introduction

Drusen are extracellular deposits accumulating between the basal lamina of the retinal pigment epithelium (RPE) and Bruch’s membrane, and are considered the hallmark of early age-related macular degeneration (AMD)^1^. Recently, a new subtype of drusen referred as ‘pachydrusen’ has been described. Pachydrusen are defined by their association with thick choroids and characterised by their appearance, distribution, and pattern of aggregation. They have well defined borders that may have a jagged margin around an area of subretinal pigment epithelial accumulation. They are also distributed throughout the posterior pole rather than accumulated at the central macula^2^. However, whether pachydrusen confer similar risk for neovascularization from AMD as soft drusen remains unclear.

The clinical significance of intermediate and large soft drusen and risk of progression to late AMD including exudative macular neovascularisation (MNV) from AMD, is well established^3,4^. Larger drusen area is a well-established association with progression to exudative MNV from AMD as detailed in the AREDs study and these findings helped create and refine the Age-Related Eye Disease Study (AREDS) severity scale^5^. This scale was subsequently validated in several other large populations studies including our own^6,7^. A subsequent study by our group also showed that larger OCT-derived drusen volume was associated with advancing AMD stages. Pachydrusen, a newly described phenotype, was not graded for specifically during AREDS and thus the natural history and clinical significance of pachydrusen is not well described. It has been suggested that eyes with pachydrusen may have a propensity to develop polypoidal choroidal vasculopathy (PCV), a subtype of neovascular AMD^8,9^. There is however a paucity of longitudinal data regarding the progression of pachydrusen as well as the progression to exudative MNV. To address this gap, we studied fellow eyes (study eyes) of patients with exudative MNV in the first presenting eye with the aim of evaluating quantitatively the longitudinal changes in clinical characteristics of pachydrusen compared to soft drusen. We hypothesized that our study eyes were more likely to progress to exudative MNV. Specifically, we analysed baseline drusen area, change in drusen area and drusenoid PED changes in these 2 groups and also assess the relationship with different subtypes of exudative MNV.

## Methods

This analysis was part of the Asian AMD Phenotyping Study, a prospective observational cohort study of Asian patients with AMD^10^. The study was approved by the Singhealth Centralized Institutional Review Board (protocol R697/47/2009) and was conducted according to the tenets of the Declaration of Helsinki with written informed consent was obtained from each patient before participation in the study. Detailed methodology for the AMD phenotyping study has been previously reported^10^. Briefly, the Asian AMD Phenotyping Study prospectively recruited consecutive treatment-naïve participants with exudative maculopathy secondary to MNV from AMD from the retinal clinic of the Singapore National Eye Centre (SNEC) since March 2010 and is still ongoing^10,11^.

Each patient was examined at baseline according to a standardized protocol^12,13^. All patients had a standardized history, clinical examination including measurement of best-corrected visual acuity, slit-lamp biomicroscopy indirect fundus examination, and underwent color fundus photography (CFP), fluorescent angiography (FA) and indocyanine green angiography (ICGA) performed with the Heidelberg Spectralis HRA (Heidelberg Engineering, Germany) or flash camera (TRC-50DX, Topcon, Japan) and Spectral domain OCT (SD-OCT) with enhanced depth imaging (EDI) mode (Spectralis, Heidelberg Engineering, Germany). Follow up interval and duration were determined by the treating physician. However, a minimum of annual review was offered to all patients.

For the purpose of this longitudinal analysis, analysis was performed on the study eye that did not exhibit any exudative MNV at patients’ first visit (Jan 2010–Dec 2013). The study eye also had to have CFP of adequate quality to assess drusen features for at least 2 years from first visit examination or at the point of development of exudative MNV. Any patients with exudative MNV in both eyes or macular scar in the study eye were excluded. Only 8 patients were excluded due to inadequate image quality for drusen assessment.

### Measurements and definition of drusen and exudative MNV

Multimodal imaging evaluation of drusen subtypes, quantitative drusen characteristics, exudative MNV subtypes and choroidal thickness (CT) was performed by trained graders from the Singapore National Eye Centre (SNEC) Ocular Reading Centre (SORC).

### Drusen subtype

Multimodal imaging using a combination of CFP and SD-OCT was used to grade the subtype of drusen. The baseline drusen subtypes graded followed published definitions based on 50° CFP centered on the fovea in a standardized digital viewing platform (ImageNet R4; Topcon Corp., Tokyo, Japan) and SD-OCT to determine the level of deposits^2^.

Soft drusen were graded as present if a collection of homogenous sub-RPE deposits that correspond to yellow-white aggregates was detected on CFP. Morphologically, soft drusen are round or ovoid and have a poorly defined border which can be tightly packed or even confluent, and may have overlying pigmentation. Pachydrusen were defined as isolated or scattered yellow-white deposits corresponding to homogenous sub-RPE material on SD-OCT. Morphologically, pachydrusen may have an ovoid or complex outer border shape, are typically scattered and isolated anywhere in the temporal vascular arcades, and do not have overlying pigment.

Pseudodrusen were graded as present if discrete subretinal accumulations (> 10) corresponding to whitish deposits on CFPs were detected. In addition, near infra-red enface and cross section SD-OCT was used to grade the presence of pseudodrusen. Eyes were classified into 2 groups. Eyes with soft drusen and eyes with pachydrusen. No eyes had both pseudodrusen and pachydrusen. Only 1 eye had soft and pachydrusen and this eye was included in the soft drusen group. Only 4 eyes had soft drusen and pseudodrusen and these eyes were classified into the soft drusen group.

### Quantitative drusen measures

#### Drusen location

Drusen location was defined as concentric rings that were centred on the fovea similar to the definition from the Wisconsin age-related maculopathy grading system with the difference where we graded the image to its visible extent and not restricted to 3000 µm^12^. Briefly the radius of the innermost circle corresponds to 500 µm in the fundus, the radii of the middle circle to 1500 µm and the outer circle with radii of > 1500 µm and beyond.

#### Drusen size

Built-in digital planimetry tools (ImageNet R4; Topcon Corp., Tokyo, Japan) were used to demarcate the area of drusen in square millimetres. The non-confluent areas of drusen were summed to yield a single value for analysis. Baseline drusen area (mm^2^) was measured on CFP at first presentation and final drusen area (mm^2^) was measured at the last available visit or last available visit prior to exudation. To account for the association of change in size with baseline^14^, the square root transformation of drusen area (SQDA) was performed for each measure. Proportion change in drusen area per year and proportion change in SQDA per year was derived from the difference between final and baseline area/SQDA divided by baseline area/SQDA over the number of years of follow up and expressed as a percentage change per year.

#### Exudative MNV

Diagnosis of exudative MNV in the first presenting eye and subsequent progression in study eyes was made by the treating physician using multimodal imaging and further classified angiographically following criteria from published literature^15–17^. These lesion characteristics were correlated to the new Consensus on Neovascular Age-Related Macular Nomenclature for Reporting (CONAN) group criteria^18^. Study eyes that eventually progressed to exudative MNV were defined as progressors and eyes that were not were defined as non-progressors.

Occurence of exudative MNV location as defined on angiography was compared to prior image to determine the location of exudative MNV in relation to drusen.

### Outcomes and statistical analysis

Only 1 eye per patient (study eye) was used in the analysis. Patients were divided into 2 groups; pachydrusen and soft drusen groups. The mean ± standard deviations of the drusen area at baseline line and final visit were calculated for each group. To analyse these changes, a paired t-test was performed and *p* < 0.05 was considered statistically significant. Percentage change per year in drusen area and SQDA was assessed by mixed-effects regression models with the drusen group as the main predictor variable after adjusting for age, follow up time, baseline drusen size and choroidal thickness.

Comparison of characteristics of eyes that developed exudative MNV and those that did not was performed, and specifically within the group that developed exudative MNV. Occurrence of PCV was compared using a logistic regression model adjusted for age, baseline drusen size, follow up time and choroidal thickness between soft drusen and pachydrusen groups.

## Results

We included 75 consecutively recruited patients that fulfilled the selection criteria. At baseline, 29 eyes had pachydrusen and 46 eyes had soft drusen. The baseline characteristics of patients in each group are summarized in Table 1. Between the two groups, there was no statistical difference in the age (mean age ± SD, 69.7 ± 9.2 years versus 66.3 ± 8.1 years, p = 0.15), gender (female n [%], 27 [61.4] versus 14 [48.2]. p = 0.15) and mean follow up time (mean years of follow up ± SD, 4.2 ± 0.7 years versus 5.0 ± 0.8 years, p = 0.52). The soft drusen group had significantly larger drusen area (4.4 ± 2.5 mm^2^ versus 1.2 ± 1.6 mm^2^, p = 0.02) and thinner choroid (221.8 ± 70.6 µm versus 283.4 ± 67.6 µm, p < 0.01) compared to the pachydrusen group. More patients were found to have PCV in their non study eye (first eye) (72.4%) in the pachydrusen group versus the soft drusen group (39.1%) and conversely less had type I and II MNV in the pachydrusen group (27.6%) versus the soft drusen group (60.8%), p = 0.02.

**Table 1:** Baseline characteristics of drusen subtype.

		Soft drusen	Pachydrusen	p-value
		n = 46	n = 29	
Age, year	Mean ± SD	69.7 ± 9.2	66.3 ± 8.1	0.15
Gender, Female	n (%)	27 (61.4)	14 (48.2)	0.15
Baseline size, mm^2^	Mean ± SD	4.4 ± 2.5	1.2 ± 1.6	0.02
Baseline SQDA, mm	Mean ± SD	1.7 ± 1.2	0.9 ± 0.6	0.02
Baseline CT, µm	Mean ± SD	221.8 ± 70.6	283.4 ± 67.6	< 0.01
Drusenoid PED, present	n (%)	10 (22.2)	2 (6.9)	0.16
**First eye nAMD subtype**				
MNV type I	n (%)	24 (52.1)	8 (27.6)	0.02
MNV type II	n (%)	4 (8.7)	0 (0)	
PCV	n (%)	18 (39.1)	21 (72.4)	

### Drusen location

**Figure 1:**
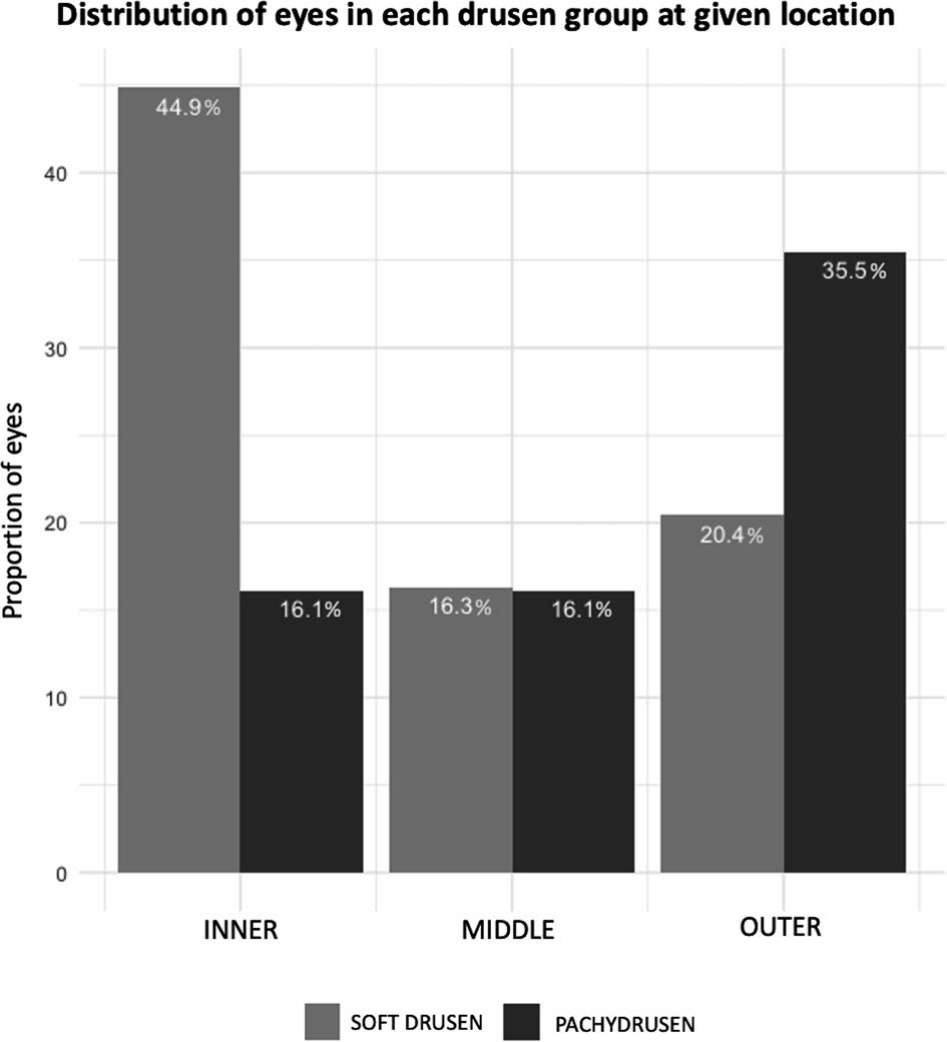
Proportion of drusen subtypes at different locations. A larger proportion of eyes with soft drusen had drusen located more centrally (within a 500 µm radius circle centred on the fovea) than in the outer areas and conversely, larger proportion of eyes with pachydrusen had drusen located in the outer ring (defined by an area bounded by an inner radius of 1500 µm to the visible extent of the photograph).

There was a significantly larger proportion of soft drusen compared to pachydrusen found in the central area (44.9% versus 16.1%, p = 0.04) and conversely a smaller proportion of soft drusen compared to pachydrusen found in the outer area (20.4% versus 35.5%, p = 0.04) (Fig. 1).

### Growth patterns according to drusen subtypes

Drusen growth patterns are summarized in Table 2. There was a larger increase in drusen size per year in soft drusen eyes compared to pachydrusen eyes (2.6 ± 0.9 mm^2^/year versus 0.4 ± 0.5 mm^2^/year, p = 0.03). To correct for baseline drusen size, we further expressed the change in drusen area as SQDA/year and soft drusen had significant increase in SQDA per year (final SQDA 2.3 ± 1.1 mm versus baseline SQDA 1.7 ± 1.2 mm, p < 0.01) but not pachydrusen (final SQDA 1.1 ± 0.7 mm versus baseline SQDA 0.9 ± 0.6 mm, p = 0.21). Finally, we also expressed the change in size over time as a proportion change od SQDA from baseline/year and soft drusen increase more compared to pachydrusen (12.0 ± 8.7% per year versus 2.2 ± 3.4% per year, p < 0.01).

**Table 2:** progression characteristics of drusen subtype.

		Soft drusen	Pachydrusen	p-value
		n = 46	n = 29	
Mean duration of follow up, years	Mean ± SD	4.2 ± 0.7	5.0 ± 0.8	0.13
Final drusen area, mm^2^	Mean ± SD	7.6 ± 7.1	1.7 ± 2.9	< 0.01
Final SQDA, mm	Mean ± SD	2.3 ± 1.1	1.1 ± 0.7	< 0.01
Drusen area change/year, mm^2^/year	Mean ± SD	2.6 ± 0.9	0.4 ± 0.5	0.03
SQDA change/year, %/year	Mean ± SD	12.0 ± 8.7	2.2 ± 3.4	0.02
Incident exudative change, n (%)	n (%)	6 (13.3)	7 (24.1)	0.38
Mean time to exudative nAMD, years	Mean ± SD	3.7 (0.9)	4.5 (1.3)	0.46
**nAMD subtype**				
Type 1 MNV	n (%)	4 (66.7)	1(14.3)	< 0.01
Type 2 MNV	n (%)	1 (16.7)	0	
Type 1 and 2 MNV	n(%)	0	0	
Type 3 MNV	n (%)	0	0	
PCV	n (%)	1 (16.7)	6 (85.7)	

### Drusen characteristics in eyes that progressed to exudative MNV

A total of 13 (17.3%) eyes developed exudative manifestations out of the 75 eyes at risk. Six were from the soft drusen group (6/46) and 7 from the pachydrusen group (7/29) (13.3% versus 24.1%, p = 0.38). The mean time to exudative MNV occurrence was 4.1 ± 1.1 years and was similar between groups (mean years (SD): soft drusen group, 3.7 ± 0.9 years and pachydrusen, 4.5 ± 1.3 years, p = 0.46). In eyes with pachydrusen, the subtype of exudative MNV was more likely manifested as PCV compared to eyes with soft drusen (85.7% versus 14.1%, p ≤ 0.01). When exudative MNV occurred they co-localised to soft drusen in all eyes of the soft drusen group. By contrast the exudative MNV co-localised to pachydrusen in only 2 of 7 eyes (29%); both of which developed the PCV subtype. This difference was highly significant (p < 0.01) (Table 2).

The characteristics of eyes that developed incident exudative MNV and those that did not (progressors and non progressors) are summarized in Tables 3 and 4. Progressors had significantly larger drusen area at baseline (2.9 ± 2.1 mm^2^ versus 0.7 ± 1.8 mm^2^, p = 0.04). About two-thirds of eyes in both groups had an increase in drusen area up to the end of follow up or visit immediately prior to exudative MNV development (69.4% versus 61.3%, p = 0.80). The proportion SQDA change per year was not statistically different in progressors compare to non progressors (8.8 ± 4.5% versus 6.0 ± 3.6%, p = 0.12).

**Table 3:** Baseline characteristics of incident exudative nAMD and stratified by drusen subtype.

		Progressor (n = 13)	Non progressor (n = 62)	p-value	Soft drusen (n = 46)			Pachydrusen (n = 29)		
					Progressor (n = 6)	Non-progressor (n = 40)	p-value	Progressor (n = 7)	Non-progressor (n = 22)	p-value
Baseline drusen size (mm^2^)	Median (IRQ)	2.9 (2.1)	0.7 (1.8)	0.04	7.1 (4.4)	1.5 (2.4)	0.03	1.2 (1.2)	0.3 (1.1)	0.1
Baseline SQDA (mm)		1.7 (1.4)	0.9 (1.3)	0.04	2.7 (1.8)	1.2 (2.1)	0.03	1.1 (0.5)	0.6 (0.7)	0.1
Baseline CT (µm)		240.0 (63.0)	242.0 (73.2)	0.908	213.0 (44.8)	229.0 (83.0)	0.64	274.0 (33.5)	282.5 (90.0)	0.33

**Table 4:** Progression characteristics of incident exudative nAMD and stratified by drusen subtype.

	Progressor (n = 13)	Non progressor (n = 62)	p-value*	Soft drusen (n = 46)			Pachydrusen (n = 29)		
				Progressor (n = 6)	Non-progressor (n = 40)	p-value	Progressor (n = 7)	Non-progressor (n = 22)	p-value*
Final drusen area	5.0 (4.2)	1.1 (2.0)	0.06	16.4 (7.5)	5.2 (4.6)	0.01	2.1 (1.8)	0.4 (0.5)	0.22
Final SQDA	2.2 (2.6)	1.0 (2.0)	0.06	4.0 (2.3)	1.5 (1.4)	0.01	1.4 (0.8)	0.6 (0.4)	0.22
SQDA change/year	8.8 (4.5)	6.0 (3.6)	0.12	10.9 (6.7)	5.1 (6.0)	0.02	3.8 (4.9)	3.2 (3.8)	0.32

*Kruskal wallis test for comparison of non-parametric variables.

**Figure 2:**
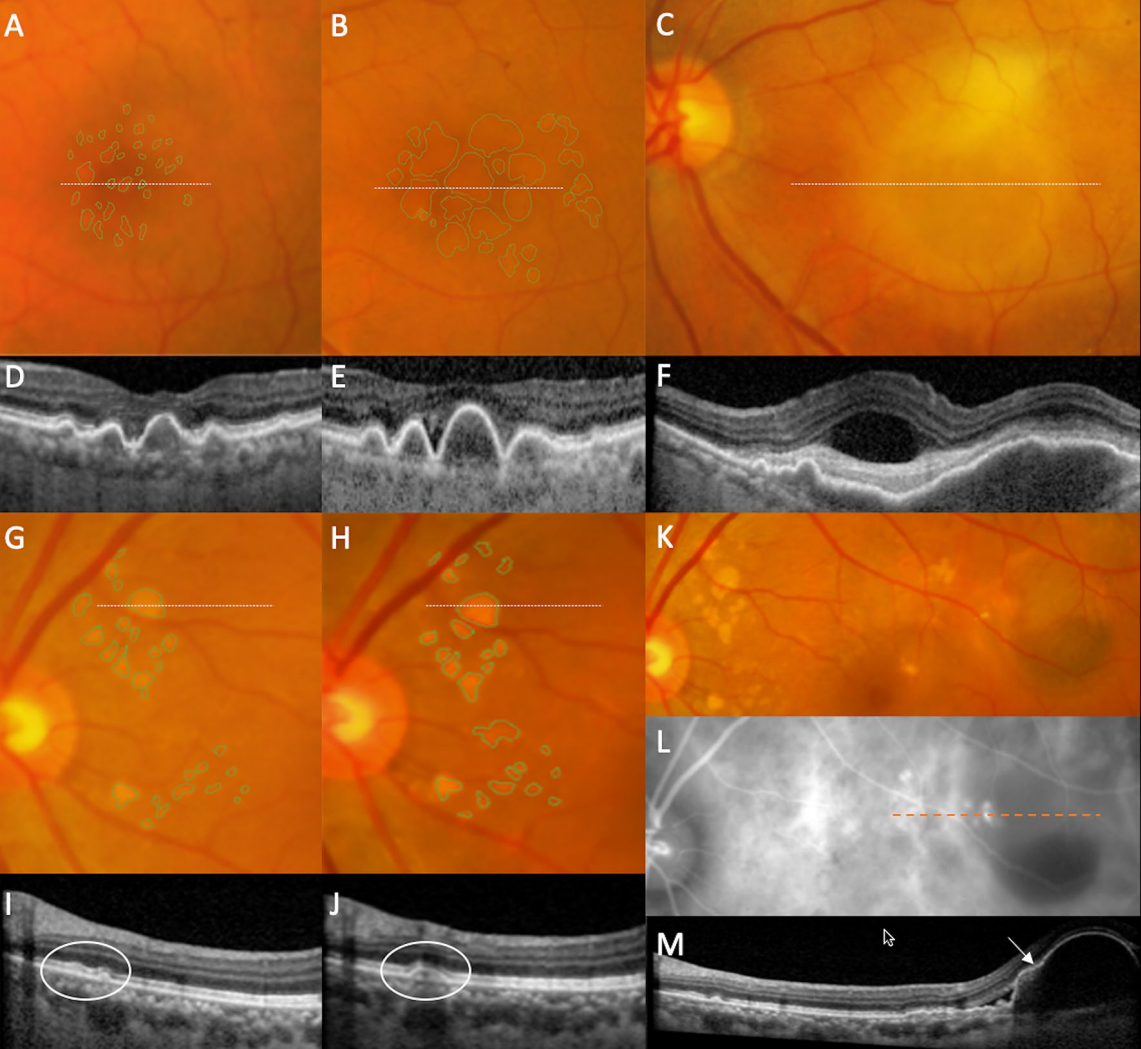
Progression of soft drusen (**A**–**F**) and pachydrusen (**G**–**M**) on color fundus photography (CFP) and optical coherence tomography (OCT) to incident exudative MNV. Location of OCT line scans through drusen of interest are indicated by dotted lines on the corresponding CFP. (**A**–**F**) In this eye with soft drusen at baseline visit (baseline total drusen area size 2.45 mm^2^), increase in size and area of drusen was noted over a follow up period of 3 years (final total drusen area size 6.16 mm^2^) (**A**,**B**). Incident exudative MNV (**C**) was noted at 10 months after previous visit (**B**). In the corresponding OCTs, there was increase in drusen height (**D**,**E**), followed by development of subretinal fluid, subretinal hyperreflective material and a large, irregular pigment epithelial detachment (PED) (**F**). (**G**–**M**) Example of progression in an eye with pachydrusen. Pachydrusen were present at baseline (**G**). These are characterized by non-confluent, ovoid or complex shapes with well-defined borders and scattered throughout the macula with no surface pigmentation. Over a follow up period of 3 years, there was mild increase the area of pachydrusen (**G**,**H**) (baseline total area size 0.51 mm^2^ to final total drusen area size 0.66 mm^2^). The corresponding OCTs through one are shown in (**I**) and (**J**). Haemorrhagic PED secondary to polypoidal choroidal vasculopathy (PCV) was noted 8 months after the last documented CFP (**K**) and confirmed on ICGA (**L**). OCT line scan through the polypoidal lesion (indicated by the arrow on OCT and orange dotted line on ICGA) (**M**). The area of exudative MNV did not colocalise to the areas of pachydrusen.

When we stratified progressors and non progressors according to baseline drusen group (Tables 3, 4), larger baseline drusen size appeared to be significantly associated with progressors in soft drusen group but not in the pachydrusen group. The proportional increase in SQDA per year between progressors and non progressors was statistically significant only in the soft drusen group (soft drusen group, 10.9 ± 6%/year versus 5.1 ± 6.0%/year, p < 0.01 compared to pachydrusen group, 3.8 ± 4.9% year versus 3.2 ± 3.8%/year, p = 0.79). An example of progression in both drusen subtype is shown in Fig. 2.

## Discussion

There is a paucity of data regarding the natural history and clinical significance of pachydrusen in terms of progression to exudative MNV. This is because pachydrusen, a relatively new entity found to be associated with pachychoroid^19–21^ was not characterized in early AMD studies such as the original Wisconsin AMD grading system which underpins the grading criteria of many longitudinal AMD studies^12,22–25^. Our longitudinal study aims to add to this knowledge by comparing the changes in clinical characteristics between soft and pachydrusen in study eyes in patients in whom the first presenting eye was diagnosed with exudative MNV. We also examined whether drusen phenotype and progression influenced the development of exudative MNV in the study eye. Our analysis highlights several notable clinical characteristics that were different between eyes with soft drusen and pachydrusen. (1) Soft drusen tended to be larger in the overall area occupied compared to pachydrusen. (2) soft drusen exhibited more rapid growth in size compared to pachydrusen in eyes that progressed to exudative MNV. (3) Exudative MNV when it occurred colocalised with soft drusen but not with pachydrusen and (4) when eyes with pachydrusen developed exudative MNV the predominant phenotype was the PCV variant.

The size of soft drusen and growth in drusen area are known to be important risk factors for development of exudative MNV^5,26^. The mechanism underpinning onset of macular neovascularisation (MNV) from drusen is thought to be related to the upregulation of VEGF from the accumulation of basal laminar deposits, basal linear deposits, membranous debris and progressive bruch’s membrane dysfunction^27,28^. Soft drusen are the clinically visible by-products of these chronic, local inflammatory events in Bruch’s membrane^29^. Enlargement of the amount and size of these components, detected as expanding drusen size, is a proxy for the increasing severity of this process which results in neovascularization and exudative MNV at that location^30^. However widespread loss of the choriocapillaris has been reported suggesting that progression to advanced AMD is not driven by regional alterations but was more likely a reflection of pathology occurring throughout the choroid^31^. It is notable that our study found differences between soft drusen and pachydrusen in terms of rates of growth, rate of progression to MNV and the phenotype of the neovascular complex. Eyes with pachydrusen that progress to exudative MNV do not exhibit rapid expansion but on the contrary have a slow growth in size and when exudative MNV occurs it does not colocalize to the site of drusen. Our findings suggest that pachydrusen may be an epiphenomenon, likely a consequence of thicker choroidal vasculature in some eyes. We did not specifically address choroidal thickness changes nor the presence of choroidal pachyvessels adjacent to pachydrusen. Because we observed that the pachydrusen retained distinct borders, showed little tendency to coalesce and that the overall area remained stable over time we believe that focal choroidal vascular influences may play a role in the pathogenesis of this feature.

A prior study reported that exudative MNV developed at the site of pachydrusen and the authors inferred a high degree of colocalisation^32^. In this afore mentioned study the authors did not comment on presence or absence of soft drusen. While we found that exudative MNV co-localise to pachydrusen, at a rate of around 29%, it was striking that when exudative MNV occurred in eyes with soft drusen the rate of colocalization was 100%. Hence we conclude that pachydrusen are less likely to be directly related to MNV formation unlike soft drusen^19^. We hypothesize that the health of the overlying RPE in pachydrusen may be different to that overlying soft drusen. In this context it is notable that in a detailed analysis of eyes with pachydrusen, Lee et al. showed that RPE undulations and pigment migration rather than the presence of pachydrusen per se increased risk of exudation^8^.

The association of pachydrusen and the PCV variant is intriguing. We recognize that PCV may have a strong bilateral component and it is likely that eyes with PCV in the first eye would develop PCV in the fellow eye, as noted in this study. Nonetheless, our findings regarding longitudinal changes of pachydrusen is novel with previous studies only reporting on the association between pachydrusen and PCV and none tracking the progression over time^21,33,34^. Current evidence suggest that different drusen characteristics are most significantly associated with different exudative MNV subtypes^8^. Patients with different subtypes of drusen may share similar genetic background and susceptibility to late AMD, while other factors such as the choroidal environment may modulate the manifestations of the sub-phenotypes expressed^34,35^.

Our results are particularly relevant to patients with unilateral exudative MNV who are being monitored for progression to bilateral disease. These findings regarding different drusen subtypes may inform the progression of the second eye to exudative MNV prompting more frequent surveillance and early treatment once early exudation is detected^36–40^.

The strengths of this study are the long follow up interval, systematic grading using multimodal imaging reducing bias. In addition, this cohort also had a well-balanced mix of patients with pachydrusen or soft drusen but not both therefore allowing for a comparison of the longitudinal changes of both subtypes and their associations. Indocyanine green angiography was performed in all eyes that progressed to exudative MNV and we therefore we were able to distinguish PCV from typical exudative MNV. This study has several limitations. Firstly, only a small number of patients developed exudative MNV. This lower rate of conversion could have been due to the relatively younger age group of the entire cohort. This is consistent with the age range of neovascular AMD patients in Asia^41,42^ and in Singapore where we previously reported a mean age of 70 years in typical neovascular AMD and 68 years in PCV in a large case controlled epidemiology study^43^. Secondly, the number of eyes with pseudodrusen was very small in this cohort, hence we were unable to report on the characteristics on this subtype of drusen. This may have also been related to a younger age group, however the overall prevalence of reticular pseudo drusen in the Asian population generally low with a range between 6 and 15%^8,34,44^. In this study we also did not use non-invasive imaging modalities such as OCTA to characterise the choroidal vasculature which may be relevant to future studies of this type. Finally, we are unable to report on the incidence of exudative MNV in this cohort due to selection bias that has arisen as a consequence of the strict eligibility criteria that we imposed on image availability. This was to ensure that we were able to characterise the longitudinal imaging features changes in this cohort which was the primary aim of this analysis.

In conclusion, this study reports similar rate of exudative MNV development in eyes with pachydrusen and soft drusen. This finding supports pachydrusen maybe an important early clinical feature in the progression to PCV subtype. While both forms of drusen exhibit an increase in size over time, soft drusen tend to increase at a significantly faster rate in eyes that progress to exudative MNV than eyes with pachydrusen. These findings fill an important gap in our understanding of choroidal features and early signs and expression of exudative MNV.
